# Outcomes after 4 years’ experience with low suction drains. Would it be safe to go drainless or low suction?

**DOI:** 10.1186/s13019-024-02824-6

**Published:** 2024-07-17

**Authors:** Mithat Fazlioglu, Walid Hammad, Deniz Piyadeoglu, Cemal Asim Kutlu

**Affiliations:** 1 Medical Faculty Department of Thoracic Surgery, Tekirdag Namik Kemal University, Tekirdag, Turkey; 2 Al-Azhar Faculty of Medicine, Cardiothoracic surgery department, Cairo, Egypt; 3 School of Medicine, Department of Thoracic Surgery, Bahcesehir University, Istanbul, Turkey

**Keywords:** Low auto-suction drain, Seal-suction test, Underwater seal

## Abstract

**Background:**

The principles of chest drainage have not changed significantly since 1875 when Bülau introduced the idea of underwater drainage tube which became a trademark of thoracic surgery. We performed a prospective, randomized trial comparing omitting pleural drain (drainless group) versus drainage with small low suction drain (drainage group) strategies of thoracic surgery when the visceral pleura remains intact. Aiming to investigate whether these approaches represent safe treatment options.

**Methods:**

A multi-center, prospective, parallel group, randomized, controlled trial enrolling patients after thoracic procedures in which visceral pleura remained intact at the end of surgery between August 2020 and September 2023. After completion of the procedure a suction-seal test was conducted on all patients. If suction-seal test was positive to confirm absence of air leak, patients were randomized to either receive low auto-suction drain as a solo pleural drain (drainage group) or not to receive drain (drainless group).

**Results:**

During the study period, 111 patients were recruited. Eleven patients had negative Suction-seal test and were excluded by inserting a traditional underwater seal. The remaining 100 patients were randomly assigned to either drainage group with low suction drain (Fig. 1) (*n* = 50) or drainless group (*n* = 50).

**Conclusion:**

The results of this study suggest that either omitting drain or inserting a low auto suction drain safely substitutes the one-way valve when the visceral pleura remains intact. Omitting drain or inserting portable small caliber drain encourages early mobilization and is associated with shorter hospital stay.

## Background

After thoracic procedures, placement of a water seal drain has been the reference standard approach for draining residual blood, pleural effusions, or pneumothorax. However, the drainage tube is often reported as the main cause of postoperative pain and interferes with the patient’s active movements, thus prolonging the duration of hospitalization and increasing medical costs [1]. Improved surgical techniques, routine use of staplers, and extensive use of modern energy devices during thoracic operations result in better control of dissected area with minimal oozing from lymphatic and blood vessels and prevention of postoperative air leak from lung parenchyma [2]. Several studies reported that either omission of chest tube placement or insertion of small low vacuum drain after various thoracic procedures is a safe and feasible approach. However, the risk for symptomatic pneumothorax (pnx) or pleural effusions (PL) requiring further reintervention remains unclear [1, 3–5].

We performed a prospective, randomized trial to compare omitting pleural drain (drainless group) versus drainage with small low suction drain (drainage group) strategies of thoracic surgery when the visceral pleura remains intact. We aimed to investigate whether these approaches represent safe treatment options.

## Methods

**Fig. 1:**
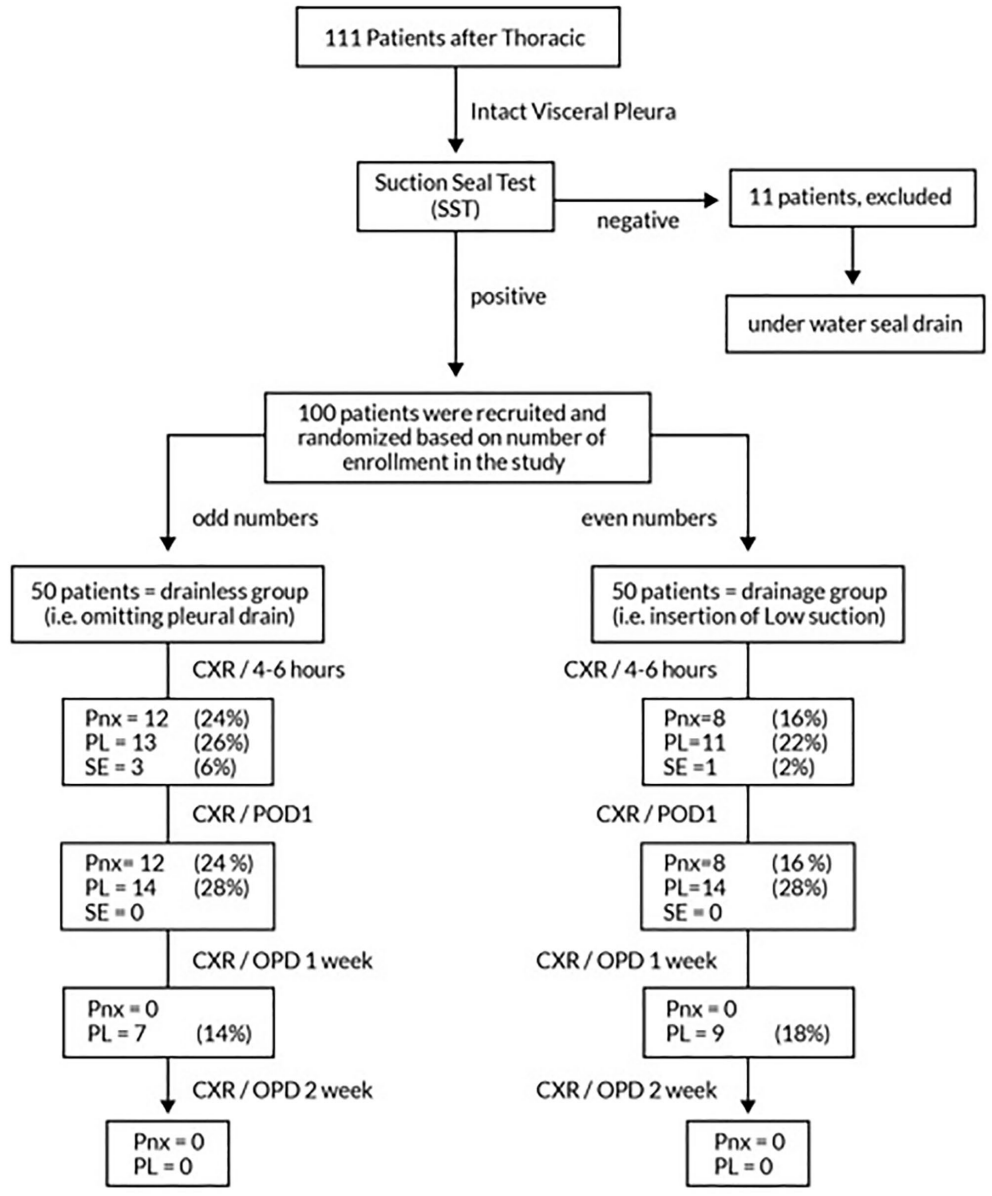
Pathway, randomization, and results of 111 patients included in the study. CXR: chest x-ray, Pnx: pneumothorax, PL: pleural effusion, SE: surgical emphysema, POD: postoperative day, OPD: outpatient department

We conducted a multi-center, prospective, parallel group, randomized, controlled trial enrolling patients after thoracic procedures in which visceral pleura remained intact at the end of surgery between August 2020 and September 2023. These procedures did not involve the lung itself, therefore the visceral pleura was not breached. After completion of the procedure a suction seal test (SST) [3] was conducted on all patients. If suction-seal test was positive to confirm absence of air leak, patients were randomized to either receive low auto-suction drain as a solo pleural drain (drainage group) or not to receive drain (drainless group) (Fig. 1). We excluded patients who had chest wall resection (> 2 ribs), accidental major hemorrhage, dense pleural adhesions, redo procedures, or removal of large mediastinal masses (defined as any mediastinal mass that cause mechanical compression of vital mediastinal structures due to the sheer weight and volume of the tumor).

### Thoracoscopic data

Whenever the VATS approach was indicated, we used KARL STORZ endoscope with 30-degree camera. For patients with pleural biopsy, we used the Uniport technique. A 2 cm skin incision was performed in the planned intercostal space and the parietal pleura was opened under direct vision. The index finger was used to lyse the possible adhesions of the lung to the chest wall, and the trocar was always inserted by means of a Kelly clamp [6].

### Confirmation of absence of air leak and sealed wound

After confirming that there was no air leakage from the treated lung evident by visual inspection of any visceral pleural breach and negative bubble test if needed. We conducted a suction seat test (SST) [3] to test for air leak either from around the drain site (i.e. unsealed system) or incidentally disintegrated visceral pleural surface. A 10 F Redivac tube was tunneled for at least one intercostal space above or below the incision level to create a valve mechanism. This tube was then connected to 250 ml bottle while the knob was deflated, and the tube remained clamped. After closing all the incisions and painting them with Dermabond skin glue, the clamps were released. The SST test is considered positive (i.e. no air leak) if the knob/container remains deflated. On the other hand, if it fails to maintain suction by inflated knob or inflate after collapse, the test is considered negative (i.e. presence of air leak or unsealed system). A negative test was conducted one more time after exclusion of unsealed system by ensuring tight entry wound, sealed connectors, and uncracked tubes. In the drainage group, the 10 F Hemovac drain was left in situ after ensuring adequate tunneling as the drain should be inserted through the same incision but undermined to a higher or lower intercostal space to create valve mechanism. This tightens the wound closure at the drain exit of the chest wall. This track can be done submuscular or subcutaneously. In the drainless group, the drain was removed, and the site was closed tightly and efficiently to ensure sealed wounds.

### Randomization

After confirmation of absence of air leak and sealed wound, Patients were randomized at a 1:1 ratio according to the patient number in the study. Patients were assigned to either the drainless or drainage group according to the sequence of patient enrolment in the study. Odd numbers were assigned to drainless group and even numbers were assigned to low suction drain.

### Postoperative monitoring and care after surgery

All patients were extubated in the operating theater and managed according to the same protocol. Postoperatively, patients were transferred to a recovery unit for a few hours and subsequently to the thoracic surgical ward. Chest X-ray (CXR) was performed at 4 to 6 h after procedure and on postoperative day 1 (POD1). We define pnx less than 5 cm as “Small pnx” and effusion that did not exceed the shadow of the 6th rib anteriorly as “Minimal effusion”. In the drainage group, the suction-seal and amount of drainage was hourly documented during the first 4 h after the operation. Thereafter and up until drain removal, the chest drain was checked at least once in every shift, three shifts per 24 h. This continuous evaluation by the surgical team aimed to early detection of suction failure and help with early decision to swap to one way valve such as underwater seal drain if suction cannot be maintained. On the surgical ward all patients followed a routine postoperative course, including checking air entry, surgical emphysema, and pain, wound, blood, antibiotic, and comorbidities management. In addition to physiotherapy and nutritional support. Drains were removed if the CXR was deemed satisfactory by primary surgeon after an independent official report of no significant Pnx or PL.

In the drainless group, CXR were performed at 4 to 6 h after procedure and repeated on postoperative day 1 (POD1). The size of residual pneumothorax was defined as the largest distance between the pleural line and the chest wall on CXR. Patients were eligible for discharge if no notable pneumothorax (less than 5 cm in diameter) was noted on serial CXR.

All patients were seen at the outpatient clinic a week after the procedure. Repeat chest x-ray was not required if breath sounds were normal on auscultation unless partial pneumothorax or pleural effusion was detected on postoperative CXR. If any radiological but clinically insignificant abnormalities were detected, a second review in two weeks’ time to follow the patients’ progression was planned.

### Statistical analysis

the outcomes of the randomly assigned 100 patients were analyzed. Continuous variables are expressed as means ± SD and compared using t tests. Categorical variables are expressed as percentages and were compared using Pearson chi-square tests. Statistical analyses were performed using SAS software (version 21, IBM, Armonk, NY).

## Results

During the study period, 111 patients were recruited. Eleven patients had negative Suction-seal test and were excluded by inserting a traditional underwater seal. The remaining 100 patients were randomly assigned to either drainage group with low suction drain (*n* = 50) or drainless group (*n* = 50) (Fig. 1).

The demographic data and the procedures undertaken were listed in Table 1. There were no significant differences between the two groups in terms of gender, age, smoking history, BMI. Age range was 4–86 years. Mild adhesion was found in 3 patients in the drainless group and 2 patients in drainage group. The visceral pleura over the pulmonary surface was not manipulated in all these procedures to avoid air leak. Types of procedures listed in Table 2. All pleural biopsies who were involved in our study (25%) were performed under general anesthesia to facilitate getting collapsed lungs and perform multiple biopsy sites. The author’s strategy is to select which patient would need biopsy under sedation or general anesthesia. These were decided on according to ASA score, planned procedure, and required position.

**Table 1:** This table demonstrates patients’ demography and the thoracic procedures performed where the vesceral pleural remained intact

Demography	Total		Drainless	Drainage	*P* value
Age	4–86		12–86	4–68	0.018
Men	55		22 (44%)	23 (46%)	0.261
Women	45		24 (48%)	21 (42%)	0.243
smoking history	67		32 (47%)	35 (52%)	0.176
BMI	19–34		26	28	0.005

**Table 2:** This table lists the thoracic procedures performed where the vesceral pleural remained intact. Trachea-oesophagial fistula (TOF), Thoracic outlet syndrome (TOS)

Procedure	Total	Drainless	Drainage	*P*-value
Pleural biopsy	25	15	10	0.003
Talc Pleurodezis	19	9	10	0.016
Mediastinal procedure	19	8	11	0.009
remova/biopsy of intrathoracic LNs	10	3	7	0.015
Exploration after thoracic trauma	6	3	3	1.000
Hematoma drainage	4	2	2	1.000
pleurectomy for Pnx	4	2	2	1.001
Diaphragmatic hernia/evantration	3	2	1	0.123
Pericardial biopsy/window	2	1	1	1.000
Rib biopsy	2	1	1	1.000
Chest wall procedure (Tumour resection, TOS repair, Pecuts repair, repair of Lung hernia)	4	3	1	0.342
TOF repair	2	1	1	1.000
Total	100	50	50	

In the drainage group (Table 3), apical pneumothorax (< 5 cm) was detected on the immediate postoperative chest x-ray in only 8 patients (16%) as shown in Table 2: the mean and SD for the sizes of pneumothorax were 0.67 ± 0.43 cm. Due to its clinical insignificance and stability on chest x-ray on POD1, regular observation was continued. On POD 1, the mean and SD for the sizes of pneumothorax had reduced to 0.59 ± 0.38 cm. The drain was removed between 6 and 56 h after the procedure. During the postoperative course, only 6 patients (12%) had the reservoir bag emptied more than once. The drain was removed by the end of POD1 in 38 patients (76%). Prolonged drainage up to 48 h was required in 8 patients (16%). Those were discharged with the drain and reviewed next morning, and the drain was removed. Minimal pleural fluid was seen on the immediate post operative x-ray in 11 patients (22%) which remained stable on the following day before discharge. Another 3 patients had their POD1 x-ray reported minimal effusion. After one week, only 9 patients (18%) had residual minimal effusion on follow up x-ray at first outpatient clinic review. Those were seen for follow up by a second out-patient visit undertaken 3 weeks from surgery with complete resolution of the effusion was detected. Only one patient (2%) in the drainage group had mild surgical emphysema. None of the patients required insertion of a chest drain or thoracocentesis. No complication related to the using Hemovac drain was reported.

**Table 3:** This table lists the average drainage time unit per day, frequanecy of reservior drainge, and secondry outcomes of either Pneumothorax or effusion during the follow up period

	Drainless	Drainage	*P*-value
Drainage time			
< 24	N/A	38 (78%)	
24–48	N/A	8 (16%)	
Emptied reservoir bag (> one)	N/A	6 (12%)	
Pneumothorax			
4–6 h (rate)	12 (24%)	8 (16%)	0.02
4–6 h (size)	0.87 ± 0.65 cm	0.67 ± 0.43 cm	0.678
POD1 (rate)	12 (24%)	8 (16%)	0.002
POD1 (size)	1.67 ± 1.31 cm	0.59 ± 0.38 cm	0.598
1 week (OPD)	0	0	
2 weeks (OPD)	0	0	
Pleural effusion (minimal)			
4–6 h	13 (26%)	11 (22%)	0.544
POD1	14 (28%)	14 (28%)	1.002
1 weeks	7 (14%)	9 (18%)	0.679
2 weeks	0	0	
Surgical emphysema	3(6%)	1 (2%)	0.183
Reintervention	0	0	
Drain insertion or reinsertion	0	0	
Readmission	0	0	

In the drainless group (Table 3), residual pneumothorax was noted in 12 out of 50 patients (24%) at 4 to 6 h; the mean and SD for the sizes of pneumothorax were 0.87 ± 0.65 cm. the number remain the same, but the size has relatively increased on POD1 CXR. The mean and SD for the sizes of pneumothorax were 1.67 ± 1.31 cm. The rate of residual pneumothorax was higher in the drainless group on CXR performed at 4–6 h and POD 1 (12 out of 50 [36%] versus 8 out of 50 [16%]; *P* = .002). However, there was no statistical significance between drainless and drainage groups for the size of Pnx on CXR performed at 4–6 h and POD1 with a *P* value of > 0.05. The rate had become comparable between groups by the time of the first outpatient follow-up visit. All pneumothoraxes were spontaneously absorbed on repeat x ray after one week without the need for any intervention in both groups. Minimal pleural fluid was seen on the immediate post operative x-ray and in 13 patients (26%) which remained stable on the following day before discharge. An additional patient had his POD1 x-ray reported minimal effusion which made the rate 14%. All of them had Talc pleurodesis. By the end of POD1, the rate of residual pleural effusions was comparable between groups with *P*-value 1.002. In one-week time, 7 patients (14%) had residual minimal effusion on follow up x-ray in outpatient clinic from the drainless group. Those were also seen for follow up by a second out-patient visit undertaken 3 weeks from surgery with complete resolution of the effusion was detected. Three patients (6%) in the drainless group had mild surgical emphysema. No patient in either group required simple needle aspiration or chest drain insertion/reinsertion, or reoperation. No patient in either group required readmission after discharge. At 1 month after surgery, all patients were discharged back to the care of their primary care.

## Discussion

This prospective, randomized, controlled trial investigated whether drainless or small low suction drain after thoracic surgery with intact visceral pleura is safe and effective. Our findings indicated that in the absence of air leak confirmed by positive suction seal test, drainless practice is safe and suggests that small residual pneumothorax, mild surgical emphysema or minimal pleural effusion do not generally represent cause for concern. Indeed, most of these findings were resolved completely at the first outpatient follow-up visit. We started using the low suction drain in 2019. Over this period, and based on the very minimal or no drainage and the permanent collapsed bag, we questioned whether the drain was necessary in the first instance.

In our previous research on 125 patients, Fazlioglu M et al. [3] suggested that low vacuum drainage systems are a feasible alternative to water seal drainage systems in the remarkable number of thoracic procedures which had the visceral pleura intact following the procedure. We concluded such drains are not required after the thoracic procedures of which air leak is not a main concern. This took us to investigate the safety of omitting a drain. We also noticed an increase in the number of published reports regarding drainless thoracic surgery in recent years [3, 5]. Despite the restricted inclusion criteria, postoperative pneumothorax/air space remains the main morbidity which reported in 59% of the patients by Castro P et al. [5]. Given the high rate of pneumothorax in their study, we only omitted the chest drain in patients who proved to have no air leak evidenced by positive suction seal test. We also believe that in the absence of air leak, low vacuum bottles provide negative pressure in the pleural cavity, which promotes efficient lung expansion and prevents fluid accumulation.

On the other hand, concerns associated with omitting chest tube drainage after thoracic procedure refer to the risk for symptomatic pneumothorax, bleeding, and pleural effusions. In this study, we selected patients with specific thoracic procedures where the visceral pleura has not been handled. Our choice of study population was based on the fact that the risk for large pneumothorax, symptomatic bleeding, and effusions is low in patients with intact visceral pleura. Although 24% of patients in the drainless group had residual pneumothorax at 4 to 6 h after surgery, none of them required reintervention, and all patients were successfully treated conservatively. Although the rate of residual pneumothorax on recovery and POD 1 radiographs were higher in the drainless group than in the drainage group, 24% and 16% respectively. There was no difference between groups by the first follow-up at the outpatient department. This observation suggests that residual air after surgery could be absorbed safely and quickly. Indeed, the rate of residual pleural effusion did not differ significantly between groups.

The results of this study suggest that either drainless or low auto suction drain safely substitutes the one-way valve when the visceral pleural remained intact and combines simplicity along with the short hospital stay. Emitting drain or inserting portable small caliber drains encourage early mobilization and is associated with lesser pain, shorter hospital stays and lower cost.

## Limitations

We acknowledge the following limitations to the study. First, this randomization was done at the time of wound closure. Second, the surgical strategy presented in our study could be considered lesser invasive thoracic procedures.

## Conclusion

The results of this study suggest that either omitting drain or inserting a low auto suction drain safely substitutes the one-way valve when the visceral pleural remained intact. Emitting drain or inserting portable small caliber drains encourage early mobilization and is associated with shorter hospital stay.

## Data Availability

No datasets were generated or analysed during the current study.

## Abbreviations

SST: suction-seal test
CXR: chest x-ray
Pnx: pneumothorax
PL: pleural effusion
SE: surgical emphysema
POD: postoperative day
OPD: outpatient department
